# Compassionate de-escalation of life-sustaining treatments in pediatric oncology: An opportunity for palliative care and intensive care collaboration

**DOI:** 10.3389/fonc.2022.1017272

**Published:** 2022-10-13

**Authors:** Andrea Cuviello, Melisa Pasli, Caitlin Hurley, Shalini Bhatia, Doralina L. Anghelescu, Justin N. Baker

**Affiliations:** ^1^ Division of Quality of Life and Palliative Care, Department of Oncology, St. Jude Children’s Research Hospital, Memphis, TN, United States; ^2^ Pediatric Oncology Education Program, St. Jude Children’s Research Hospital, Memphis, TN, United States; ^3^ Division of Critical Care Medicine, Departments of Pediatric Medicine and Bone Marrow Transplantation and Cellular Therapy, St. Jude Children’s Research Hospital, Memphis, TN, United States; ^4^ Department of Biostatistics, St. Jude Children’s Research Hospital, Memphis, TN, United States; ^5^ Division of Anesthesiology, Department of Pediatric Medicine, St. Jude Children’s Research Hospital, Memphis, TN, United States

**Keywords:** palliative care, palliative sedation therapy, dexmedetomidine, propofol, pediatric oncology, symptom management, end of life

## Abstract

**Context:**

Approximately 40%-60% of deaths in the pediatric intensive care unit (PICU) are in the context of de-escalation of life-sustaining treatments (LSTs), including compassionate extubation, withdrawal of vasopressors, or other LSTs. Suffering at the end of life (EOL) is often undertreated and underrecognized. Pain and poor quality of life are common concerns amongst parents and providers at a child’s EOL. Integration of palliative care (PC) may decrease suffering and improve symptom management in many clinical situations; however, few studies have described medical management and symptom burden in children with cancer in the pediatric intensive care unit (PICU) undergoing de-escalation of LSTs.

**Methods:**

A retrospective chart review was completed for deceased pediatric oncology patients who experienced compassionate extubation and/or withdrawal of vasopressor support at EOL in the PICU. Demographics, EOL characteristics, and medication use for symptom management were abstracted. Descriptive analyses were applied.

**Results:**

Charts of 43 patients treated over a 10-year period were reviewed. Most patients (69.8%) were white males who had undergone hematopoietic stem cell transplantation and experienced compassionate extubation (67.4%) and/or withdrawal of vasopressor support (44.2%). The majority (88.3%) had a physician order for scope of treatment (POST – DNaR) in place an average of 13.9 days before death. PC was consulted for all but one patient; however, in 18.6% of cases, consultations occurred on the day of death. During EOL, many patients received medications to treat or prevent respiratory distress, pain, and agitation/anxiety. Sedative medications were utilized, specifically propofol (14%), dexmedetomidine (12%), or both (44%), often with opioids and benzodiazepines.

**Conclusions:**

Pediatric oncology patients undergoing de-escalation of LSTs experience symptoms of pain, anxiety, and respiratory distress during EOL. Dexmedetomidine and propofol may help prevent and/or relieve suffering during compassionate de-escalation of LSTs. Further efforts to optimize institutional policies, education, and collaborations between pediatric intensivists and PC teams are needed.

## Introduction

Each year in the United States, approximately 20,000 children die, and beyond the first year of life, the majority of those deaths are due to accidental trauma, congenital anomalies, malignancy, or intentional injury (1, 2). Although the distribution of the causes of pediatric deaths has not changed significantly over several decades (2), the events leading up to death have. The advancement of medical treatments and evolution of pediatric critical care has altered the progression of several pediatric disorders and increased invasive interventions during the end-of-life (EOL) period (1, 3).

Life-sustaining treatments (LSTs), such as mechanical ventilation and vasoactive support, play a substantial role in supporting patients during EOL; nevertheless, they may contribute to symptom burden and suffering (4, 5). Palliative care (PC) as a medical subspecialty focuses on improving quality of life (QOL) and decreasing suffering through symptom management, psychosocial support, and advanced-care planning (5–7). Integration of PC throughout the disease trajectory and within the pediatric intensive care unit (PICU) may improve outcomes and has become increasingly accepted, yet such services remain underutilized (8, 9).

In pediatric oncology, advancements in hematopoietic cell transplantation (HCT) and immunotherapy have increased overall survival and critical care needs in this population have risen accordingly (10). In fact, nearly 40% of pediatric oncology patients are admitted to the PICU at some point during - therapy, and more than half of these patients require multiple PICU admissions (3, 11). Additionally, mortality rates for pediatric oncology patients admitted to the PICU are notably 4-fold greater than those for the general pediatric population who require intensive care (12, 13). Patients undergoing HCT can have further increases in mortality risk that are associated with respiratory failure requiring mechanical ventilation and prolonged PICU stays (>15 days) (10, 14).

However, regardless of diagnosis, prognosticating in the PICU is difficult and comes with substantial uncertainty (15). When faced with a terminal prognosis, families can find themselves having to make difficult decisions regarding LSTs (16). In this context, involvement of PC specialists during PICU admissions can improve shared medical decision making, decrease parental regret, and assist with bereavement (8, 17). Nearly 40%-60% of pediatric deaths in the PICU occur after a decision is made to withdraw LSTs (1, 18–20); however, little is known about how parents and families arrive at that decision. One study suggests that, in most cases, medical professionals initiate conversations about compassionate de-escalation of LSTs, and consensus is reached between medical teams and families after 1-2 meetings (21). The role of PC during this time remains poorly defined, with the potential for missed opportunities to provide improved QOL, support decision making, and give psychosocial support to these families.

In general, literature on compassionate de-escalation of LSTs is limited and it is rarely specific to pediatric oncology. To address this gap in the literature, we conducted a retrospective review of deceased pediatric hematology/oncology patients treated at an academic hospital over a 10-year period, with the goal of describing this patient population and their EOL experiences.

## Methods

An Institutional Review Board–exempt, retrospective review was performed of pediatric hematology/oncology patients treated at St. Jude Children’s Research Hospital between April 1, 2011, and January 1, 2021. This date range defines a period when patients would have all pertinent data for this study placed into the electronic medical record system.

For the purposes of this study, the term LSTs was defined as patients who required mechanical ventilation and/or vasoactive support. Patients requiring non-invasive respiratory support eventually progressed to intubation and mechanical ventilation and thus are also represented in this definition. Inclusion criteria consisted of age <25 years, a confirmed hematologic or oncologic diagnosis, death occurring in the PICU, and patients who had de-escalation of LSTs, specifically compassionate extubation, withdrawal of vasopressor support, or both. Other LST withdrawal was not assessed due to the complexity of recognizing the rationale for withdrawal in our electronic medical record.

Two study members (AC and MP) performed data extraction in a systematic fashion using a data dictionary to ensure consistency. Data collected included demographics [age at diagnosis, sex, race/ethnicity, religious affiliation, date of diagnosis, date of death, age at time of death (TOD)], disease characteristics (primary oncology or hematology diagnosis, stage of disease, presence of relapse or recurrence, history of hematopoietic cell transplantation, type of transplant, cancer-directed treatment within the last month and week of life, infectious complications during last admission), EOL care characteristics (date of PC first contact, goal of care at the time of PC consultation, number of PC visits, hospice enrollment and date if applicable, date of first pain service consultation, number of pain service visits, intubation status at TOD, withdrawal of LSTs, cardiopulmonary resuscitation on day of death (DOD), Do Not Resuscitate status) and medications used for symptom control within 24 hours of death. Of note, the presence of symptoms (i.e., pain, anxiety, nausea, etc.) was ascertained through daily progress note documentation. Discrepancies were reviewed by both study members until a consensus was reached.

Descriptive statistics of the data included frequency (percent), mean ± standard deviation (SD), and median [Max, Min]. SAS (version 9.4, SAS Inc.) was used for all analyses.

## Results

### Patient demographics

A total of 721 patients died during the study period: 244 deaths occurred in the inpatient setting, and 107 occurred in the PICU. Of those patients who died in the PICU, 43 (40.2%) had withdrawal of LSTs and thus met the study’s criteria. The majority (58.1%) were male, primarily white (69.8%), and their mean age at diagnosis was 5.54 years (median [Min, Max] = 3 [0.02, 18]) (**Table 1**).

**Table 1:** Demographics of 43 pediatric hematology/oncology patients who received compassionate de-escalation of life-sustaining treatments.

Demographic characteristic	Frequency (%)^a^
Sex	
* Female*	18 (41.2)
* Male*	25 (58.1)
Age at diagnosis (years)	
* Mean* (*SD*)	5.54 (5.67)
* Median* [*Min*, *Max*]	3 [0.02, 18.0]
Age at TOD (years)	
* Mean* (*SD*)	7.56 (6.52)
* Median* [*Min*, *Max*]	7 [0.17, 23]
Race/Ethnicity	
* White/Non-Hispanic*	30 (69.8)
* Black*	6 (14.0)
* White/Hispanic*	3 (7.0)
* White, South/Central American*	1 (2.3)
* Multiple races*	1 (2.3)
* Hispanic*	1 (2.3)
* Declined*	1 (2.3)
Status Post Hematopoietic cell transplantation (allogeneic)	
* Yes*	24 (55.8)
* No*	19 (44.2)

a Data represents the number of patients (%), unless otherwise indicated.

Max, maximum value; Min, minimum value; No., number of; SD, standard deviation; TOD, time of death.

### Disease characteristics

Sixteen (37.2%) patients had acute lymphoblastic leukemia, and 11 (25.6%) had acute myelogenous leukemia. Evidence of relapsed disease was found in 24 (55.8%) patients, and 23 (53.5%) had undergone allogeneic HCT (**Table 2**).

**Table 2:** Disease characteristics of 43 pediatric hematology/oncology patients who received compassionate de-escalation of life-sustaining treatments.

Disease characteristic	No. patients (%)
Primary Oncology Service	
* Bone Marrow Transplantation*	23 (53.5)
* Hematology*	3 (7.0)
* Leukemia*	7 (16.3)
* Neuro-Oncology*	8 (18.6)
* Solid Tumor*	2 (4.7)
Primary Diagnosis	
* Acute lymphoblastic leukemia*	16 (37.2)
* Acute myelogenous leukemia*	12 (27.9)
* Atypical teratoid rhabdoid tumor*	5 (11.6)
* Rhabdoid tumor*	1 (2.3)
* Severe aplastic anemia*	1 (2.3)
* Dyskeratosis Congenita*	1 (2.3)
* Ependymoma*	1 (2.3)
* Evans syndrome*	1 (2.3)
* Fanconi anemia*	1 (2.3)
* Wilms tumor*	1 (2.3)
* Glioblastoma*	1 (2.3)
* Retinoblastoma*	1 (2.3)
* Medulloblastoma*	1 (2.3)
Disease relapse	
* Yes*	24 (55.8)
* No*	19 (44.2)

### End-of-life care

Of the 43 patients included in the study, 25 (58.1%) received cancer-directed therapy during their last month of life, and 20 (46.5%) received it during their last week (**Table 3**). Among the patients requiring compassionate de-escalation of LSTs, 24 (55.8%) underwent compassionate extubation, 14 (32.6%) had withdrawal of vasoactive support, and 5 (11.6%) experienced both (**Table 4**).

**Table 3:** End-of-life characteristics of 43 pediatric patients undergoing compassionate de-escalation of life-sustaining treatments.

End-of-life characteristic	Frequency (%)^a^
Cancer-directed treatment during the last month of life	
* Yes*	25 (58.1)
* No*	18 (41.9)
Cancer-directed treatment during the last week of life	
* Yes*	20 (46.5)
* No*	23 (53.5)
No. Palliative Care visits	
*Mean* (*SD*)	14.2 (14.7)
*Median* [*Min*, *Max*]	9 [0, 61]
*No. patients who met Palliative Care on DOD*	8 (18.6)
No. Pain Service visits	
*Mean* (*SD*)	6.98 (25.2)
*Median* [*Min*, *Max*]	0 [0, 118]
*No. patients who did not interact with the Pain Service*	36 (83.7)
Goal of Care	
* Cure*	24 (55.8)
* Comfort*	10 (23.2)
* Life prolongation*	4 (9.3)
* Poor prognosis^b^*	2 (4.7)
* Life prolongation and comfort*	1 (2.3)
* Not documented*	2 (4.7)
Enrolled in Hospice	
* Yes*	5 (11.6)
* No*	38 (88.3)
POST in place	
* Yes*	38 (88.3)
* No*	5 (11.6)
CPR administered on DOD	
* Yes*	1 (2.3)
* No*	42 (97.6)
Time between POST and DOD (days)	
* Mean* (*SD*)	13.9 (61)
* Median* [*Min*, *Max*]	1 [0, 373]
Time between intubation and DOD (days)	
* Mean* (*SD*)	1 (2.78)
* Median* [*Min*, *Max*]	0 [0, 14]
Examination for brain death^c^	
* Yes*	5 (11.6)
* No*	38 (88.4)

a Frequency indicates the number of patients (%), unless otherwise indicated.

b Poor prognosis, while not a true “goal of care” was part of the documentation template for the goals of care section.

c One patient with no formal examination for brain death was noted as having brainstem disruption.

DOD, day of death; CPR, cardiopulmonary resuscitation; EOL, end of life; Max, maximum value; Min, minimum value; No., number of; PICU, pediatric intensive care unit; POST, physician order for scope of treatment; SD, standard deviation; TOD, time of death.

**Table 4:** Frequency of life-sustaining treatments withdrawn at the end of life and symptoms experienced.

Life-sustaining treatment	Frequency (%)
Intubated at the TOD	
* Yes*	15 (34.9%)
* No*	28 (49.1%)
Vasopressors withdrawn before DOD	
* Yes*	19 (44.2%)
* No*	24 (55.8)
**Symptoms at EOL in the PICU**	
Respiratory distress	
* Yes*	42 (97.7%)
* No*	1 (2.3%)
Anxiety/agitation	
* Yes*	34 (79.0%)
* No*	9 (21.0%)
Pain	
* Yes*	40 (93.0%)
* No*	3 (7.0%)

DOD, day of death; EOL, end of life; PICU, pediatric intensive care unit; TOD, time of death.

Regarding EOL characteristics, 38 (88.3%) patients had a physician order for scope of treatment (POST - DNaR) in place before death, and only 1 (2.3%) patient received cardiopulmonary resuscitation on the DOD (**Table 3**). POSTs were completed an average of 13.9 days (median [Min, Max] = 1 [0, 373]) before DOD (**Table 3**).

### Palliative care consultation

PC was consulted for 42 (97.6%) patients, and the average number of PC visits was 14.2 (median [Min, Max] = 9 [0, 61]) (**Table 3**). Of note, 8 (18.6%) patients received PC consultation on the DOD; however, for the remainder, PC was involved approximately 2 weeks before the DOD. For many patients and families, the goal of care remained cure, despite PC involvement, and only 5 (11.6%) patients enrolled in hospice.

### Pain management consultation

Only 6 (14.2%) patients received pain service consultation, and the average number of pain service visits was 6.9 (median [Min, Max] = 0 [0, 118]) (**Table 3**). Those who received pain service consultation did so on average 4.3 months before the DOD and the consult was for pharmacological pain management. None of the patients received interventional pain modalities for pain management at the end of life.

### Medication management

Complex medication regimens addresses the symptom burden at the EOL. Respiratory distress (97.6%, n=42), pain (93.0%, n=40), and anxiety/agitation (79.1%, n=34) were the most commonly reported symptoms experienced at the EOL (**Table 4**).

**Figure 1:**
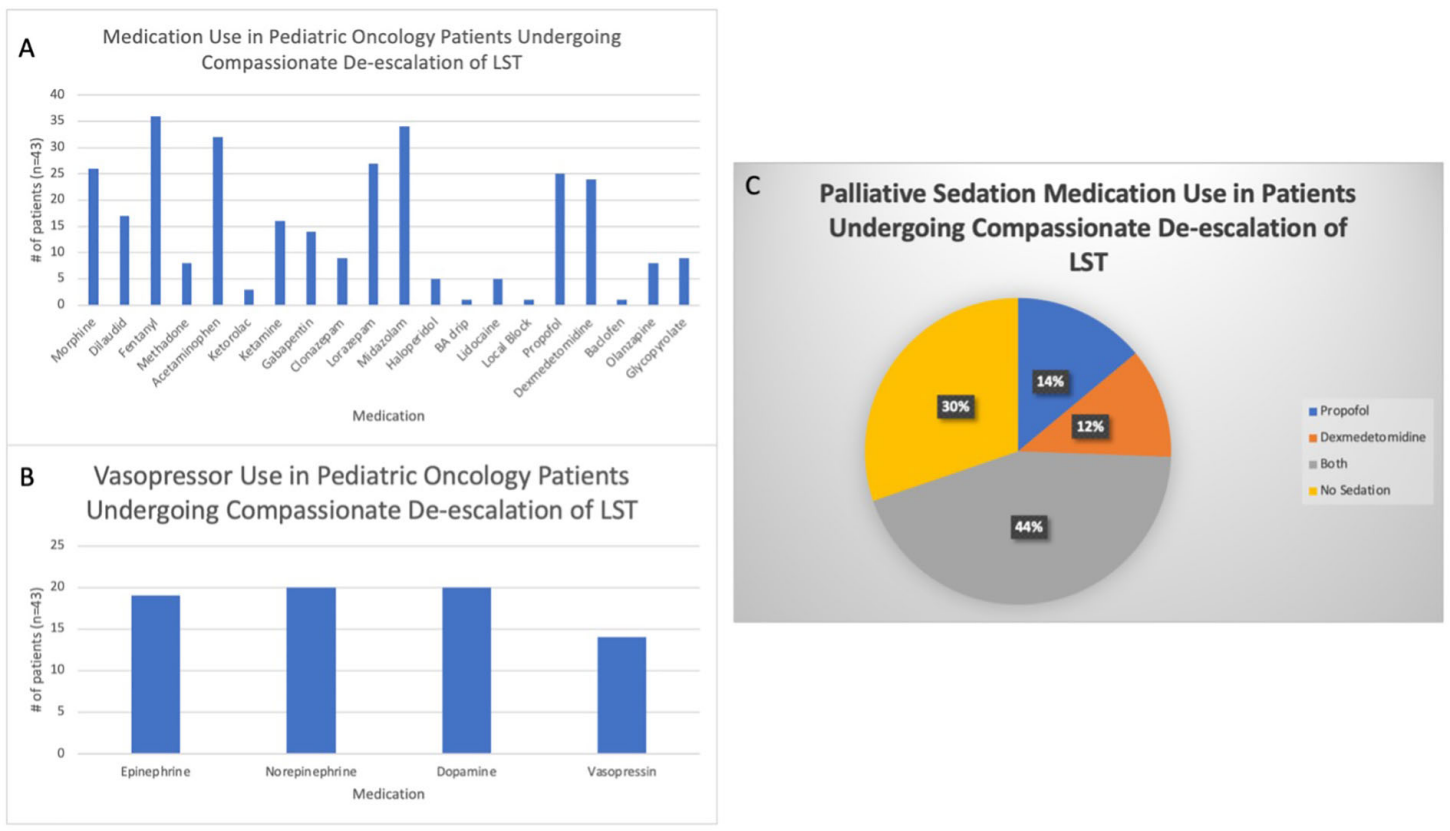
Medications used to treat pediatric oncology patients during compassionate de-escalation of life-sustaining treatments (LSTs). **(A)** All medications prescribed to the study population to treat symptoms at the end of life. BA= Benadryl/Ativan. **(B)** Vasopressor medications used to treat hypotension in the study cohort. **(C)** Use of palliative sedation therapy, specifically dexmedetomidine and propofol, during the end of life.

All patients received one or more opioid medication to manage their symptoms. Midazolam (79.1%, n=34) and lorazepam (62.8%, n=27) were the two benzodiazepines most often prescribed (**Figure 1A**). For vasopressor and inotropic support, norepinephrine and dopamine were most commonly prescribed (46.5%, n=20), followed by epinephrine (44.2%, n=19) and vasopressin (32.6%, n=14) (**Figure 1B**). Sedative agents, such as propofol (14.0%, n=6), dexmedetomidine (11.6%, n=5), or both (44.2%, n=19), were administered (**Figure 1C**).

## Discussion

Our study examined de-escalation of LSTs for pediatric hematology/oncology patients at the EOL in the PICU setting, a topic that has not been summarized in more than a decade and is often generalized for all pediatric patients (13). The need for intensive care, and by default implementation of LSTs, such as mechanical ventilation and vasopressor or inotropic support, for pediatric hematology/oncology patients is associated with increased mortality risk (13, 22–25), especially for patients undergoing HCT (10, 14). Additional factors associated with poor overall survival of patients admitted to the PICU include multisystem organ dysfunction (24, 26–28) and sepsis (13). Despite advances in the treatment of childhood cancers, the risk for therapeutic toxicities, including death, and the prevalence of suffering at the EOL remain prominent (29).

We found that across all deaths that occurred in the PICU during the study period, 40.2% of patients experienced compassionate de-escalation of LSTs, specifically either removal of vasopressor/inotropic support, or compassionate extubation, or both. This is consistent with the literature, which suggests that approximately 40%-60% of all PICU deaths occur after removal of LSTs (18–20). The majority of patients had leukemia, which was not surprising, as leukemia is the most common oncologic diagnosis in children (30). Additionally, HCT is performed primarily for hematologic malignancies at the study institution, as noticed in over half of the study cohort, and is associated with significant risk for treatment-related toxicity, including death (31–34). Despite medical advances in the treatment of childhood cancer, 1 in 5 patients will still succumb to their disease (35), and it is well documented that the EOL period can be complicated by physical, psychosocial, emotional, and spiritual suffering (5, 31, 35–37).

From the onset of diagnosis, oncologists facilitate important conversations surrounding disease status and therapeutic options and partner with pediatric intensivists to care for patients when intensive care is required and potentially during the EOL period (38). One way to help minimize the suffering experienced by patients during the EOL in the PICU setting and improve QOL may be through early engagement and collaboration with PC teams. Our study showed that early collaboration between intensivists and PC teams is feasible and it is supported by the literature. Evidence throughout the literature suggests several opportunities for interfacing and collaboration between intensivists and PC team, to improve the EOL experience of patients, their families, and even medical teams, through assistance with advanced-care planning, shared medical decision making, particularly when faced with decisions about de-escalation of care and symptom management, which may include palliative sedation therapy (PST) (3, 7, 32, 39). PST is defined as, “the use of sedative medications to relieve intolerable and refractory distress by the reduction in patient consciousness” (40–44). Over the last several years, acceptance of early integration of PC in the realm of pediatric oncology and PICU care has grown with institutions using the PICU admission as a trigger for PC consultation (38, 45–49).

All but one patient in our study engaged with the PC team. The PC team was involved an average of 2 weeks before the DOD, allowing for time to build rapport and trust with families. This is important for the patient and family, as well as all medical teams, as this time enables providers to gain a better understanding of what is important to a patient and family and what their goals may be. For example, most of the families reported a goal of cure for their child, which may explain the finding that approximately half of our study population received cancer-directed treatment during the last month and/or week of life. By extension, this information helps the medical team provide support and guidance to patients and families through shared medical decision making. Although the literature is rich on the topic of shared medical decision making, little is known about how families come to the decision to forgo LSTs (50–53). One prospective study found that parents initiate a conversation about compassionate de-escalation of care in about 25% of cases, and that consensus can be reached between medical teams and families after one meeting in about 50% of cases (21). Future exploration of the timing of decision making, withdrawal of LSTs, use of PST, and barriers to family consensus are recommended.

In addition to augmenting discussions on de-escalation of LSTs, the PC team can work with the medical teams to facilitate conversations of advanced-care planning, specifically in providing an extra layer of support for families and medical staff. In some instances, these conversation may include facilitating death in the home setting and coordinating hospice services, and for many patients in an acute ICU setting these conversations simply revolve around creating a calm, loving EOL period with family members at the bedside and forgoing cardiopulmonary resuscitation that may incur suffering (1, 54, 55). More than 80% of our cohort had a Do Not Resuscitate order in place before death, and only 1 patient received cardiopulmonary resuscitation. Additionally, the average time between Do Not Resuscitate decisions and death was approximately 14 days, which we hypothesized allowed families time to discuss all options and make a well-informed, goal-centric decision for their child and family. In contrast, approximately 20% of the patients in our cohort met the PC team on the DOD. We believe that unexpected acute changes to signify a high likelihood of death and consultation occurring as the team was preparing to remove LSTs most likely contributed to this delay and poses a potential opportunity for practice improvement. As PC encompasses a holistic approach to caring for patients and families, this concept becomes increasingly important during points of high patient acuity in one’s care journey, especially when PICU care is required.

Previous research suggests the PICU admission as a time point for consideration in engaging PC teams (56, 57) and optimizing PC integration in the PICU setting. One way this may be accomplished is through an embedded model in which PICU staff members are identified and trained to be PC champions; a model that currently exists with success in some PICUs, as well as pediatric critical care fellowship programs across the nation (48). Training would include PC course work and subspecialty clinical rotation experience, with the goal of increasing awareness and education of ICU staff about PC services available to patients and families (48). Prospective studies and qualitative data would help determine the most effective way for PC teams to support patients and families during EOL in a PICU.

When death becomes inevitable, many families and caregivers begin to hope for a “good death” for their child (58, 59). The concept of a good death looks different to each family unit, and in some instances, compassionate de-escalation of LSTs has been requested to occur in the home, an option that may not be considered by healthcare professionals (4, 60–62). Many parents describe a high symptom burden at the EOL and state this as a major contributor to their child’s suffering (29). Specifically, pain, dyspnea, fatigue, and anxiety are commonly reported by patients and noted as a source of suffering by parents (7, 29, 36). Our finding of pain, anxiety/agitation, and respiratory distress being present for nearly all patients in our study further supports this. Traditional symptom management, with medications and psychological coping behaviors, are often enough to alleviate suffering, but what happens when they are not? In some cases, advanced adjuvant medications for pain can be employed (lidocaine or ketamine infusions), or interventional strategies (e.g., nerve blocks or neuraxial blocks), and even implants, such as epidural catheters, can be employed (63–67).

In rare cases, suffering persists despite all interventions, and PST is an effective tool for refractory suffering at the EOL (40–44). PST practices are variable in pediatric oncology, and medication choices for implementation of PST are evolving (40, 68, 69). Propofol and dexmedetomidine are utilized more commonly for this intervention (68–71).

Dexmedetomidine and propofol are commonly used in the PICU setting (72). However, we took a closer look at what medications were used within 24 hours of the TOD to decipher how often patients received medication regimens like those used in PST practices. Many patients were actively receiving propofol, dexmedetomidine, or both at the TOD. We hypothesize that these medications are often used for sedation while the patient receives mechanical ventilation and/or for symptom control before de-escalation of care and were continued to avoid withdrawal and ensure adequate symptom management until the TOD. An observational study of death in the PICU completed more than 2 decades ago demonstrated similar findings of sedative and analgesic use after de-escalation of LSTs in the PICU (1). It also noted 13% of medical professionals were dissatisfied with the EOL care provided and felt that the level of medication administration was inadequate for symptom management (1). However, the patient population in that study encompassed all of pediatrics and was not focused on pediatric hematology/oncology. Propofol or dexmedetomidine administration in our study may not have been intended for PST, but it remains a fascinating finding and offers an opportunity for increased education, awareness, and implementation of PST practices in the PICU. Of the 13 patients who did not receive propofol or dexmedetomidine, four had one formal documented brain death exam and died prior to a second, confirmatory exam, and one patient had documented compression of the brainstem. It is also important to note that medications, such as opioids, benzodiazepines, antiemetics, muscle relaxants, and gabapentinoids, still have an important role in symptom management at the EOL and many patients in this study required these medications as part of their symptom management.

Pain medicine specialists and anesthesiologists are an additional resource for patients, families, and medical teams mediating EOL symptoms. Our study showed that after compassionate de-escalation of LSTs, most pediatric oncology patients who died in the PICU did not involve the pain service in their care. Collaboration between the PC team and pain specialists can help optimize traditional strategies, incorporate interventional tactics (e.g., nerve blocks), and initiate PST if warranted (40).

Overall, this study builds on the limited literature on pediatric oncology patients facing de-escalation of LSTs at the EOL. We highlight opportunities for further PC integration to help optimize shared decision making, advanced-care planning, and symptom management at the EOL in the PICU. Future studies characterizing how families decide to compassionately de-escalate LSTs and prospective studies analyzing symptom burden, relief, and interventions specifically surrounding PST practices and drugs like propofol and dexmedetomidine, would be most informative.

This study had several limitations. First, it represents the experience of a single institution that sees a focused patient population with a higher level of patient acuity and death. Second, retrospective data collection relies on precise documentation; therefore, information, such as timing of discontinuation of a medication or LST, and specifics around decision making are not always easily identified. As such, this study design limits our ability to draw conclusions on causality or intent of medication use (i.e., propofol for PST) and necessitates the need for future prospective investigations.

## Conclusion

Pediatric hematology/oncology patients admitted to the PICU have increased risk of mortality, and especially when LSTs are necessary, early integration of PC may be beneficial. It remains unclear how families decide to compassionately de-escalate LSTs, how this decision may affect suffering at the EOL, and how medication practices in the PICU may incorporate concepts of PST. Collaborations between oncologists, intensivists, and PC specialists may help optimize QOL and minimize suffering. Furthermore, prospective and qualitative studies in this realm and increased educational awareness of EOL interventions, including the use of PST, are needed.

## Data availability statement

The raw data supporting the conclusions of this article will be made available by the authors, without undue reservation.

## Author contributions

All authors listed have made a substantial, direct, and intellectual contribution to the work and approved it for publication.

## Funding

This work was funded by the American Lebanese Syrian Associated Charities (ALSAC). As a participant in the Pediatric Oncology Education Program at St. Jude Children’s Research Hospital, MP was supported, in part, by R25 CA23944 from the National Cancer Institute. The content of this paper is solely the responsibility of the authors and does not necessarily represent the official views of the National Institutes of Health.

## Acknowledgments

We would like to acknowledge the support of the pediatric palliative care and pain management experts for their care to our patients, as well as the patients who participated in our study.

## Conflict of interest

The authors declare that the research was conducted in the absence of any commercial or financial relationships that could be construed as a potential conflict of interest.

## Publisher’s note

All claims expressed in this article are solely those of the authors and do not necessarily represent those of their affiliated organizations, or those of the publisher, the editors and the reviewers. Any product that may be evaluated in this article, or claim that may be made by its manufacturer, is not guaranteed or endorsed by the publisher.

## Abbreviations

DOD, day of death; EOL, end of life; HCT, hematopoietic cell transplantation; LST, life-sustaining treatment; PC, palliative care; PICU, pediatric intensive care unit; POST, physician order for scope of treatment; PST, palliative sedation therapy; QOL, quality of life; TOD, time of death.
